# Characteristics of pediatric multiple sclerosis: A tertiary referral center study

**DOI:** 10.1371/journal.pone.0243031

**Published:** 2020-12-02

**Authors:** Blažo Nikolić, Nikola Ivančević, Ivan Zaletel, Branislav Rovčanin, Janko Samardžić, Jasna Jančić

**Affiliations:** 1 Clinic of Neurology and Psychiatry for Children and Youth, Belgrade, Serbia; 2 Institute of Histology and Embryology “Aleksandar D. Kostić”, Belgrade, Serbia; 3 Faculty of Medicine, University of Belgrade, Belgrade, Serbia; 4 Center for Endocrine Surgery, Clinical Center of Serbia, Belgrade, Serbia; 5 Institute of Pharmacology, Clinical Pharmacology and Toxicology, Belgrade, Serbia; 6 Division of Paediatric Pharmacology and Pharmacometrics, University of Basel Children's Hospital, Basel, Switzerland; Instituto Cajal-CSIC, SPAIN

## Abstract

**Objective:**

The present study represents one of the largest series of pediatric multiple sclerosis (PedMS) in Western Balkan region. This is the first study aimed to evaluate the characteristics of PedMS in the Serbian population.

**Methods:**

This retrospective study on 54 PedMS, aged 7–17 years, was performed at the Clinic of Neurology and Psychiatry for Children and Youth in Belgrade, Serbia, a tertiary center for the diagnosis and treatment of children with neurological and psychiatric diseases.

**Results:**

Female to male ratio was 37 (68.5%): 17 (31.5%). Family history of MS was noted in 9.3% and autoimmune diseases in 24.1% patients. Co-occurring migraine was in 7,4%. Monofocal onset of disease was present in 77.8% patients. The most common initial symptoms were optic neuritis (37%), sensory disturbances (31.5%), motor deficit (24.1%), cerebellar (18.5%) and brainstem lesions (16.7%), pain (9.3%), acute disseminated encephalomyelitis like symptoms (1.9%), and hearing loss (3.7%). Visual evoked potentials were pathological in 75.9% of patients. Oligoclonal bands were positive in 68.5% of patients. Magnetic resonance imaging showed periventricular (94.4%), infratentorial (77.8%), juxtacortical and cortical changes (55.6%) and changes in the cervical spinal cord (33.3%). The median EDSS score was 2.0.

**Conclusion:**

Our cohort significantly differs from the literature data regarding more frequent occurrence of optic neuritis, hearing loss as a first symptom, the relapsing-remitting course of the disease, higher proportion of early onset of disease, presence of co-occurring migraine and the frequent occurrence of epilepsy and other autoimmune diseases in the family.

## Introduction

Multiple sclerosis (MS) is a chronic, neurodegenerative, demyelinating, autoimmune and multifactorial disease of the central nervous system (CNS) [1]. It is one of the most common causes of non-traumatic disability in young adults [2]. It usually occurs between the 2^nd^ and 4^th^ decade of life, however it is diagnosed rarely in individuals older than 50, and in children under 18 years of age, when the diagnosis is referred as pediatric multiple sclerosis (PedMS) [3]. PedMS accounts for 2.5–10% of the total number of patients with MS, and it is most commonly diagnosed in adolescence around the age of 15 [4–6]. Early onset of PedMS can be seen in children younger than 12 years of age, but it is extremely rare, occurring in only 1% of patients [5]. Similar to the adult MS, the etiology of PedMS, has not been fully elucidated; however, it is known to be a multifactorial disease as a result of numerous genetic interactions and environmental factors [1]. According to previously published data, a positive family history is present in more than 6% of patients with PedMS, with female sex predominance and female to male ratio of 2:1 up to 3:1 [7].

The course of the disease is different in PedMS patients compared to patients with MS that begins after 18 years of age. The most prevalent form of the disease is the relapsing-remitting (RR) form of the disease, while primary-progressive (PP) and secondary-progressive (SP) PedMS are extremely rare [4]. The diagnosis of PedMS can be based on the revised McDonald's criteria, but only for patients older than 11 years of age [8]. According to the new diagnostic criteria for MS, it is also possible to make a diagnosis based on MRI and clinical presentation after the first attack of the disease [8]. The diagnostic criteria, accepted in 2007 and subsequently updated in 2013 by the International Pediatric Multiple Sclerosis Study Group, may be used for all patients younger than 18 years of age [9, 10].

In recent years, PedMS has attracted great attention from physicians and researchers due to its epidemiological, etiological and pharmacological specificity. So far, few studies, focused on clinical characteristics and the course of the disease in PedMS, have been published with limited number of patients [11]. The present study represents one of the largest series of PedMS in the region, and it is also the first study aimed to evaluate the characteristics of PedMS in the Serbian population.

## Methods and materials

The retrospective study on 54 PedMS patients aged 7–17 years was performed at the Clinic of Neurology and Psychiatry for Children and Youth in Belgrade, Serbia, a tertiary center for the diagnosis and treatment of children with neurological and psychiatric diseases. We analyzed patient documentation between January 2012 and December 2018. The study included all patients with confirmed diagnosis of PedMS based on the diagnostic criteria from the International Pediatric Multiple Sclerosis Study Group [9, 10]. Patients who did not meet the diagnostic criteria or who met the criteria for clinically isolated syndrome or radiologically isolated syndrome were not included in this study.

The study analyzed the following data: gender, patient age and symptoms at the onset of the disease, interval between first and second attack of the disease, total number of relapses during the follow-up, clinical course of the disease defined as relapsing-remitting, primary or secondary progressive, personal history and family history of multiple sclerosis and co-occurring autoimmune diseases as well the presence of epilepsy in patients and their families. Additional diagnostic data was analyzed, such as Extended Disability Status Scale (EDSS), relapse rate, brain and cervical spinal cord magnetic resonance imaging (MRI) findings, as well as paraclinical findings of cerebrospinal fluid (CSF) oligoclonal bands and visual evoked potential (VEP) results. The study also included treatment data for all patients.

### Statistical analysis

The study results are presented by using descriptive statistics (absolute numbers, frequencies, percentages, mean and standard deviation, median and range). Fisher's Exact test and Chi squared test were used to test associations between the EDSS score and neurological lesions present in our patients. The differences were considered significant p<0.05.

All participants or their parents obtained written informed consent during hospitalization at the Clinic. The study was approved by the Ethics Committee of the Clinic of Neurology and Psychiatry for Children and Youth, (No. 21–3312.). Data for this study were collected retrospectively from medical history.

## Results

### Demographic characteristic

A total of 54 PedMS patients were analyzed, with a mean age of 16.42 ± 1.73. The study group consisted of 37 (68.5%) female and 17 (31.5%) male patients. The average age of onset of the disease was 14.30 ± 2.69 years. The gender ratio (female:male) in our study group was 2.2:1 and in patients younger than 12 years the gender ratio was 1:1. The average follow-up period was 25.11 months (range: 3 to 96 months). Early-onset (before 12 years of age) was present in 10 patients (18.5%), and late-onset (≥ 12 years) was noted in 44 patients (81.5%) patients.

There were 53 (98.1%) Caucasian patients and 1 (1.9%) patient of Arab descent in the analyzed patient group. Family history of MS was noted in 5 (9.3%) patients, while a certain number of patients showed positive family history for autoimmune diseases (13 patients, 24.1%) and epilepsy (8 patients, 14.8%). Co-occurring diseases, such as autoimmune diseases and migraine, were present in 11 (20.4%) and 4 (7.4%) patients, respectively.

### Clinical course and symptoms

**Fig 1:**
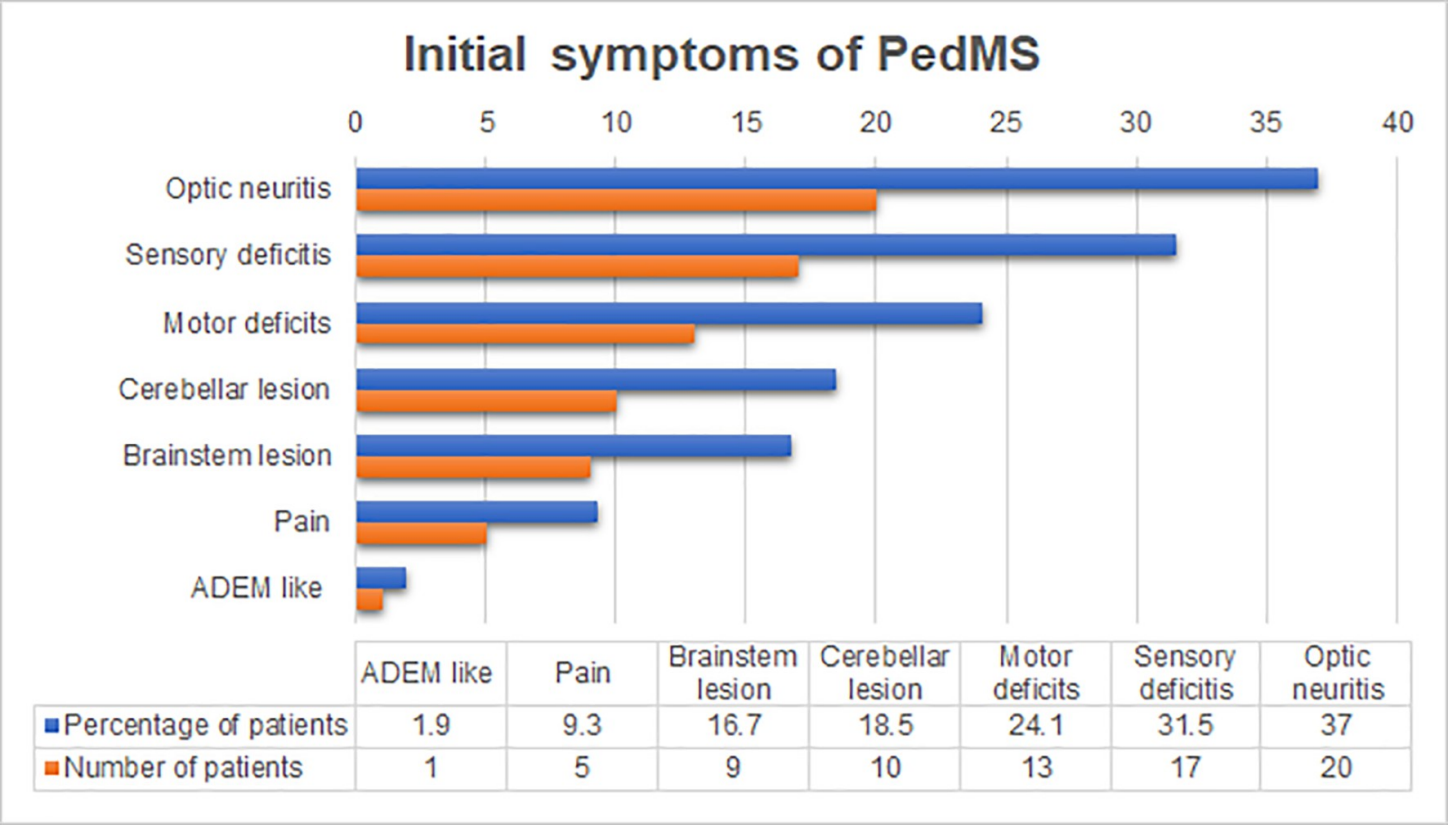
The frequency of initial symptoms in patients diagnosed with PedMS. Optic neuritis was the most frequent initial symptom, present in more than 35% of diagnosed cases, while certain symptoms such as ADEM like symptoms and hearing loss were present in less than 5% of cases. Abbreviations: PedMS–pediatric multiple sclerosis; ADEM—acute disseminated encephalomyelitis.

The mean duration of the disease at the follow-up was 25.11 ± 24.24 months (range 3 to 96 months). The average period between the first attack and relapse was 13.07 ± 18.83 months (range: 1 to 90), while the average number of relapses until 18 years of age was 3.26 ± 1.99 (range: 2 to 11 relapses). Monofocal onset of disease was present in 42 (77.8%) patients, while 12 (22.2%) patients had multifocal onset. The most common initial symptoms (Fig 1) of the disease were optic neuritis followed by sensory and motor deficit, cerebellar and brainstem lesions, pain, as well and acute disseminated encephalomyelitis (ADEM) like symptoms. Hearing loss, which represents one of the rare symptoms of brainstem lesions, was noted in 2 (3.7%) patients.

**Fig 2:**
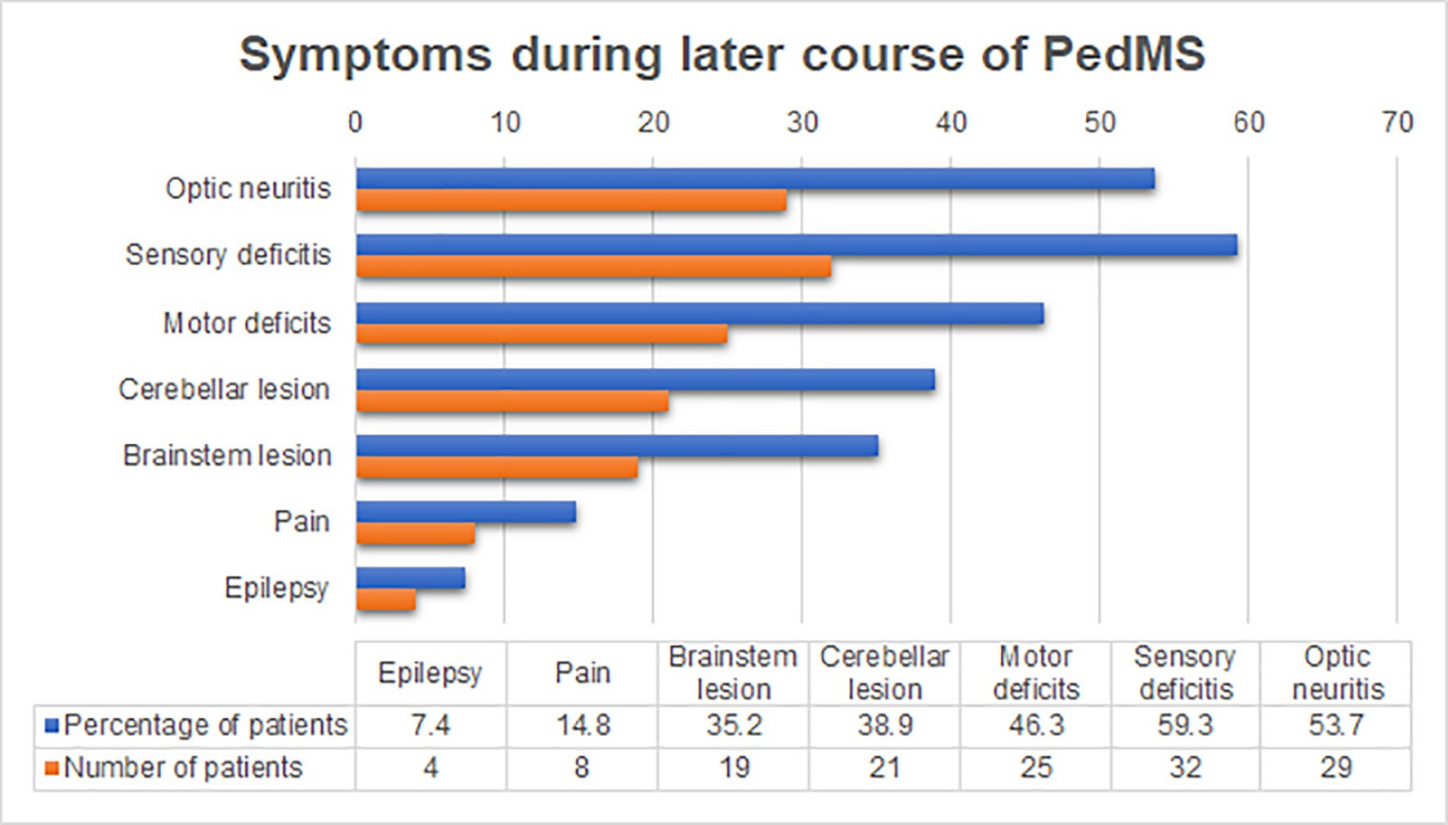
The frequency of symptoms in later course of PedMS. Sensory symptoms were the most frequent symptom, present in almost 60% of patients. Epilepsy, which was not present as an initial symptom, was diagnosed in 4 patients. Abbreviations: PedMS–pediatric multiple sclerosis.

In the later course of the disease (Fig 2) the dominant symptoms were sensory deficits, followed by optic neuritis and motor deficits. Hearing loss occurred in 4 (7.4%) patients with brainstem lesions. All patients had the RR course of the disease, while PP or SP forms were not present. Also, none of our patients had malignant forms of PedMS with a fatal outcome.

### Diagnostic procedures

Cerebrospinal fluid (CSF) and serum were analyzed for oligoclonal IgG bands in all patients. 37 patients (68.5%) had positive oligoclonal IgG bands, while no patients had parallel bands in both the CSF and the plasma. VEP was performed at the beginning of the disease in all patients with pathological findings present in 41 (75.9%) patients.

MRI changes were noted according to diagnostic criteria, which showed periventricular lesions (51 patients, 94.4%), infratentorial changes (42 patients, 77.8%), juxtacortical and cortical changes (30 patients, 55.6%) and changes at the level of the spinal cord (18 patients, 33.3%). EDSS was monitored regularly throughout all neurological controls. The EDSS score at the end of follow-ups was Median 2.0 (range: 0 to 3.0). A total of 6 (11.11%) patients had EDSS score 0 (Table 1). Patients with motor deficit statistically had significantly higher EDSS (p < 0.001). There is a statistically significant association in the incidence of EDSS scores relative to the back log of brain stem function (p < 0.05). No statistically significant association was observed, neither in patients with early-onset disease and EDSS score (p < 0.391), nor in patients with positive CSF oligoclonal bands and EDSS (p < 0.279).

**Table 1:** Expanded Disability Status Scale score in patients with pediatric multiple sclerosis.

EDSS score	Number of patients / Percentage of patients
**0**	6 (11.1%)
**1**	14 (25.9%)
**1.5**	6 (11.1%)
**2**	16 (29.6%)
**2.5**	7 (13.0%)
**3**	5 (9.3%)
**Total No.**	54 (100%)

EDSS, Expanded Disability Status Scale.

All patients had RR disease course, without any patients with PP or developing SP forms of the disease during the 7 years of follow-up.

### Therapy

All of the patients received a high dose of corticosteroids (20–30 mg/kg) as initial therapy, while 36 (66.7%) patients continued with the corticosteroid therapy in decreasing doses. Therapeutic plasma exchange (TPE), was administered to 5 (9.3%) patients after limited efficacy of prolonged high-dose corticosteroid treatment, with a relatively good recovery. None of the patients with relapse were treated with intravenous immunoglobulins.

Immunomodulatory therapy was administered to 13 (24.1%) patients. There were 2 (3.7%) patients on interferon beta1b therapy, glatiramer acetate was administered in 2 (3.7%) patients, interferon beta 1a in 6 (11.1%) and fingolimod in 4 (7.4%) patients.

## Discussion

According to Jeong et al., [12] in the period from 1965 to 2018, only 19 studies were published with clinical characteristics of PedMS patients. This is the first study on a larger number of patients suffering from PedMS in Serbia and Western Balkan region, focusing on their epidemiological and clinical characteristics. Most patient features in our PedMS group are similar to European studies; however, there are important results that differentiate our study from other published data.

The prevalence of PedMS in Serbia is 4.4/100 000, while the prevalence in the entire Serbian population is 0.77/100 000. Other PedMS studies showed incidence ranging from 0.13/100,000 to 1.24/100,000 [7] and prevalence of 5.5 to 26.92 [4]. Our results showed lower prevalence in comparison to the population of Sardinia [13] and Kuwait [14] and similar to Dutch [15] and Slovenian [16] studies. We analyzed 54 patients over a 7-year period. These are the results of a single tertiary center; however, it is a retrospective study thus the results should be considered according to such limitation. The overall female to male gender ratio was 2.2:1, a proportion similar to other European studies [17], which may indicate the potential influence of sex hormones and puberty in the etiology of PedMS [18]. However, a study examining the gender ratio of Hispanic Americans found a female to male ratio of 0.88/1 [19]. The average gender ratio in patients younger than 12 years was 1:1, which is consistent with the data from other published studies [18, 19].

PedMS studies have a different upper limit for age groups: 16 years [17, 20–22], 18 years [9, 16, 23, 24] and a recently published systematic review even included patients up to 19 years of age [12]. Due to a small number of published studies, these methodological differences can make a bias when analyzing and comparing results of different studies. Our study has followed the recommendations of the International Pediatric Multiple Sclerosis Study Group [9] in which PedMS is the defined as a disease that occurs before the age of 18. The average age at the onset of disease for our patients corresponds to other studies that reported an average onset from 13 years to 16.5 years [16, 19, 23], although some studies have reported an average onset from ages 11 to 13 years [25]. Early onset of disease is also similar to other studies, where this group of patients represents 15 to 20% of the patients [11, 23]. On the other hand, our study differs in comparison to other studies since we had 5.56% of children under the age of 10 years, while other studies have reported only up to 1% of these patients [20].

All of our patients had the RR course of PedMS. Certain studies have also registered the PP course in pediatric and adolescent patient population in about 3% of the cases [17]. A positive family history of MS existed in 9.3% of patients, while other studies reported results ranging from 6 to 8% [26], or most extremely from 2 to 19% [16, 27]. In our PedMS group, 24.1% of patients had positive family history for other autoimmune diseases. Our results show a higher prevalence of other autoimmune diseases in the family. All patients were screened for autoimmune diseases that can lead to a similar clinical finding. The higher number of reported family members may be the result of direct questions to parents about other autoimmune diseases in the family, such as autoimmune thyroid disease, type I diabetes mellitus, rheumatoid arthritis, etc.

Epilepsy was also registered in family members of 14.8% of the patients. Migraine was present in 7.4% of the patients; however, due to the lack of data, it could not be compared with other PedMS studies. In adult MS up to 20% of the patients had migraine [28].

Monofocal onset of the disease in our study is consistent with other studies, which reported monofocal onset in 54% to 90% patients, usually after puberty [5, 29]. However, a number of studies have reported multifocal onset of disease in 50 to 70% of the cases, and mostly before puberty [26]. The most common initial symptoms are similar to other studies [21, 30, 31], which also reported sensory deficits followed by loss of vision, motor deficit, lesions of the cerebellum and brainstem [5, 9, 17]. Pain was registered during the initial attack in 9.3% of patients and 3.7% had hearing loss as an initial symptom, which was previously reported in only one case [32]. The diagnosis of epilepsy was present in 7.4% of patients, while 1.9% of patients had seizures, which did not fulfill the diagnostic criteria for epilepsy. This result is similar to other studies which reported that epilepsy occurs in 5% to 16% of cases [17, 33].

We found a slightly higher percentage of positive oligoclonal IgG bands (68,5%), compared to the majority of studies where the percentage of positive findings ranged from 40% to 60% and only a few studies have reported up to 80% of patients with positive oligoclonal bands [4, 16, 23, 34]. According to the International Pediatric Multiple Sclerosis Study Group [9, 10] oligoclonal bands are not yet included in the current diagnostic criteria for PedMS, though a positive finding of CSF oligoclonal bands is important for the diagnosis of PedMS [35]. PedMS studies have shown that positive finding of CSF oligoclonal bands are less common in children than in adults with MS [4, 35]. Our study had a similar incidence of ADEM like onset of the disease as well as certain other studies [23, 36, 37]. In this retrospective study, we have included all of our patients with ADEM like onset of the disease who had a subsequent attack of the disease which is typical of MS based on its clinical and radiological characteristics.

The role of spectrum of myelin oligodendrocyte glycoprotein (MOG) antibody encephalitis, which has begun to stand out as a separate entity in recent years, is attracting increasing attention from researchers [38]. Testing our patients for MOG antibody-associated encephalomyelitis/encephalitis was implemented since 2017. and it is now a part of all patient testing at the beginning of the disease when there is a suspicion of CNS demyelinating disease. Before 2017, the differential diagnosis pointed to the possibility that some patients had a MOG antibody encephalitis, but we were not able to carry out the adequate testing. Bearing in mind that the patients previously were not tested for MOG antibody encephalitis, it is possible that some patients were positive, but the clinical course of the disease in our patients was most consistent with PedMS. Differential diagnoses were also considered and have excluded the possibility of diseases which may have similar symptoms to inflammatory, infectious, metabolic, neurodegenerative and vascular disorders. Lumbar puncture and determination of oligoclonal bands in CSF was performed in our patients only during the first attack at the onset of disease. In the later stages of the disease and in relapses, we did not perform lumbar puncture due to the invasiveness of the diagnostic method in childhood. In other PedMS studies, differences in the percentage of oligoclonal bands varies depending on the age of patients at the onset of disease [39]. In PedMS patients who didn’t have a positive finding of oligoclonal bands in the CSF at the onset of disease, a repetition of the CSF analysis should be considered later in the course of the disease. VEP findings were abnormal in 75.9% of patients at the onset of the disease, while in other studies 50% to 79% of patients had abnormal VEP [17, 23]. VEP can help us detect subclinical lesions and then recover the visual pathway, by detecting signal propagation. The visual system and the optic tract are often damaged in PedMS patients, both clinically and subclinically. In addition to neuroophthalmological examinations, we tried to have an objective evidence of visual impairment. We have taken into account the following pathological values in PedMS patients: increased latency, increased latency with morphological abnormalities of a major component, absence of a major component and lower amplitudes. In our study, VEP abnormalities were present in the optic neuritis eyes and also in the non-optic neuritis eyes. All patients who had optic neuritis had VEP abnormalities as well; however, there were patients who did not have a medical history and clinical optic neuritis, but abnormal VEP, in 21 (38.8%) patients. Thus, the finding of abnormal VEP in a significant number of patients with PedMS may represent a positive basis for VEP to be reestablished as an early diagnostic criteria in PedMS for space dissemination [40]. The results of MRI findings are similar to recently published studies [17, 23]. Our patients also had a highly active demyelination and inflammation (T1-weighted and T2 lesion loads) on MRI. Relatively low EDSS in our patients indicates the importance of adequate disease relapse therapy as well as immunomodulatory therapy [41], although they have had frequent relapses and highly active demyelination and inflammation on MRI. The results are consistent with previously published studies [17, 23].

Relapses of the disease in all patients were treated with high doses of corticosteroids for 3–5 days and 66.7% of them continued with corticosteroid therapy using decreasing doses after day 5. Our results correspond to other studies that reported treatment with high doses of corticosteroids and continuation with corticosteroid therapy at decreasing doses [42, 43]. Plasma therapeutic replacement was administered after the ineffectiveness of high doses of corticosteroids, with relatively good outcome. Some studies [44] suggest that relapses in PedMS patients may also be treated with intravenous immunoglobulins, but we did not have a single case during the follow-up. Interferon and glatiramer acetate constitute the first-line therapy, whereas second-line therapy is teriflunomide, fingolimod, dimethyl fumarate, natalizumab, rituximab, and mitoxantrone [45]. Most of our patients (75.9%) had a natural course of the disease, while only a small number of patients (24.1%) were subjected to immunomodulatory therapy. An explanation for the low percentage of patients on immunomodulatory therapy could be the late introduction of immunomodulatory therapy due to regulatory issues in Serbia. Namely, before 2018, patients received immunomodulatory therapy only in clinical trials. Over the last 2 years, PedMS patients have had priority for the immunomodulatory therapy provided by Health Insurance Fund. During our study we did not have any serious side effect associated to immunomodulatory therapy.

## Conclusion

This is the first study on PedMS in Serbia and Western Balkan. Most characteristics are similar to the other European studies; however, our cohort significantly differs from the literature data regarding more frequent occurrence of optic neuritis, hearing loss as a first symptom, the RR course of the disease in all patients, higher proportion of early onset of disease, presence of co-occurring migraine and more frequent occurrence of epilepsy and other autoimmune diseases in the family. According to our results, pathological VEP findings could be a useful diagnostic tool in some circumstances. More studies are needed to address the clinical features of PedMS in the Western Balkans and in the world, aiming to improve the quality of life of children and adolescents suffering from MS. We strongly support the early introduction of immunomodulatory therapy in PedMS.
